# Efficacy of ^18^F‐Fluoro‐2‐Deoxyglucose Positron Emission Tomography as a Predictor of Treatment Response to Neoadjuvant S‐1 + Oxaliplatin Chemotherapy for Gastric Cancer

**DOI:** 10.1002/cnr2.70190

**Published:** 2025-04-24

**Authors:** Naoki Urakawa, Shingo Kanaji, Ryuichiro Sawada, Yasufumi Koterazawa, Taro Ikeda, Hitoshi Harada, Hironobu Goto, Hiroshi Hasegawa, Kimihiro Yamashita, Takeru Matsuda, Yoshihiro Kakeji

**Affiliations:** ^1^ Division of Gastrointestinal Surgery, Department of Surgery Kobe University Graduate School of Medicine Kobe Japan

**Keywords:** gastric cancer, neoadjuvant chemotherapy, positron emission tomography

## Abstract

**Background:**

Neoadjuvant chemotherapy is widely recognized as the established treatment for advanced gastric cancer. However, predicting its efficacy before surgery remains challenging.

**Aim:**

The present study aimed to evaluate the effectiveness of 18F‐fluoro‐2‐deoxyglucose positron emission tomography (FDG‐PET) as a predictor of treatment response to the S‐1+Oxaliplatin regimen (SOX).

**Methods and Results:**

Thirty patients who underwent gastrectomy following neoadjuvant SOX between January 2021 and July 2023 were included. Patients underwent FDG‐PET pre‐ and postsurgery. The maximum standardized uptake value (SUVmax) from FDG‐PET was examined in relation to histological tumor response and prognosis. SUVmax decreased significantly after chemotherapy in all patients (*p* < 0.001), especially in those with Grade 1a, 2, and 3 tumors (*p <* 0.05). SUV reduction increased stepwise with the histological response grade. Optimal cut‐off values for the percentage decrease in SUVmax (ΔSUVmax) predictive of histologic efficacy were identified as 53% (area under curve 0.855, *p =* 0.0018) for Grade 1b or higher and 75% (area under curve 0.806, *p* = 0.0044) for Grade 2 or higher. Patients with ΔSUVmax > 50% had improved recurrence‐free survival (*p =* 0.027).

**Conclusion:**

FDG‐PET may be useful as a predictor of treatment response in neoadjuvant SOX therapy for gastric cancer. The determination of the optimal ΔSUVmax value may enhance the precision of histological tumor response prediction.

## 1 Introduction

Gastric cancer (GC) is the fifth most prevalent cancer worldwide, with a poor prognosis [1]. The effectiveness of neoadjuvant chemotherapy (NAC) for patients with GC and esophagogastric junction cancer (EGJC) has been demonstrated in clinical trials. In Western countries, the standard therapy for these patients is NAC with fluorouracil, leucovorin, oxaliplatin, and docetaxel (FLOT) [2]. Surgery and postoperative adjuvant chemotherapy remain the mainstay of treatment in Asia, which has the highest number of GC patients, although clinical trials of NAC, such as SOX and docetaxel plus S‐1 (DOS), are currently underway [3, 4, 5, 6, 7, 8]. Although NAC is an effective treatment for both GC and EGJC, its efficacy is difficult to predict before treatment initiation. Serum tumor markers such as carcinoembryonic antigen (CEA) and carbohydrate antigen 19‐9 (CA19‐9) have been investigated as predictors of chemotherapy response, but it is not clear whether changes in these markers can predict the efficacy of chemotherapy on the primary tumor [9]. Histological tumor response to chemotherapy is one of the most important determinants of the efficacy of NAC [10]. Although histological tumor response is associated with the prognosis of patients with GC and EGJC, it can only be determined after NAC followed by gastrectomy.

18F‐fluoro‐2‐deoxyglucose positron emission tomography (FDG‐PET) is useful in diagnosing tumor progression in various cancers. Computerized tomography (CT) and magnetic resonance imaging (MRI) are generally used to evaluate the size of tumors and the presence or absence of metastasis. PET‐CT is not only useful for these evaluations but also for evaluating tumor viability, and its usefulness has been reported in various carcinomas. However, some reports have indicated that its efficacy is compromised in adenocarcinomas of GC and EGJC [11, 12, 13]. In our previous study, the maximum standardized uptake value (SUVmax), which indicates the degree of FDG accumulation in the primary tumor, was identified to be an indicator for predicting the depth of GC and lymph node metastasis. The efficacy of FDG‐PET for GC and EGJC remains controversial [14, 15, 16].

The present study aimed to clarify the utility of FDG‐PET for preoperative determination of the efficacy of NAC for GC and EGJC. We examined the association of changes in SUVmax with histological tumor reactivity, divided into six grades based on the Japanese classification of gastric carcinoma and prognosis [8].

## 2 Patients and Methods

### 2.1 Patients

The study included 30 patients with surgical resection after preoperative chemotherapy SOX for GC or EGJC at Kobe University Hospital between January 2021 and July 2023, who underwent a PET scan at the initial visit and after NAC. The inclusion criteria for patients in this retrospective study were as follows: surgically resectable adenocarcinoma, clinical depth of T2 or more, lymph node metastasis, preoperative chemotherapy SOX, Eastern Cooperative Oncology Group performance status 0 or 1, and PET‐CT before and after preoperative chemotherapy. The exclusion criteria included metastasis to other organs at the time of initial diagnosis, synchronous cancer, a history of chemotherapy for other cancers, palliative resection, and other chemotherapy regimens. There were 31 patients who met these criteria, but one patient was lost to follow‐up during chemotherapy, so the number of patients was 30. GC and EGJC were diagnosed using total‐body CT and esophagogastroscopy biopsy. Clinical lymph node metastases were assessed as positive at 8 mm or more. The histologic and clinicopathological assessments, comprising the evaluation of histological tumor response, were conducted in accordance with the 15th Japanese Classification of Gastric Carcinoma established by the Japanese Gastric Cancer Association [10]. Approval for this study was obtained from the Kobe University Ethics Committee (Approval No. B230038). As this was a retrospective study, the Kobe University IRB waived the need to obtain informed consent.

### 2.2 Neoadjuvant Chemotherapy

The NAC regimen was SOX with S‐1 (body surface area < 1.25 m^2^, 40 mg × 2; body surface area between 1.25 and 1.49 m^2^, 50 mg × 2; and body surface area ≥ 1.50 m^2^, 60 mg × 2) + oxaliplatin (130 mg/m^2^), and three courses were administered at 3‐week intervals. If HER2‐positive, the regimen was combined with trastuzumab (6 mg/kg). Chemotherapy‐related toxicities were assessed based on the Common Terminology Criteria for Adverse Events (CTCAE, version 5.0). The discontinuation or postponement of chemotherapy was determined by the following criteria: white blood cell count ≤ 1000/mm^3^; platelet count ≤ 50,000/mm^3^; neutrophil count ≤ 500/mm^3^; or Grade ≥ 3 nonhematological adverse events.

### 2.3 ^18^F‐Fluoro‐2‐Deoxyglucose Positron Emission Tomography

All patients underwent whole‐body FDG‐PET/CT scans at the initial visit and after the completion of NAC. Whole‐body FDG‐PET was conducted by using a PET scan (Philips Allegro; Philips Medical System, Best, Netherlands). A luminescence scan was conducted around 1 h after intravenous injection of approximately 222–333 MBq (6–9 mCi) of FDG. The emission PET scans were reconstructed using a row‐action maximum likelihood technique, resulting in a matrix size of 128 × 128. Following the PET scan, participants underwent a CT scan at 120 kV and 80 mA. The PET and CT images were combined using automatic image‐fusion software (Syntegra; SUN Microsystems). The maximum standardized uptake value (SUVmax) was quantified using FDG‐PET/CT. SUV was calculated using the following formula (human density: 1 g/mL): SUV = organizational radioactive concentration [kBq/mL]/(administered radioactivity [MBq]/body weight [kg]). The maximum value was taken as SUVmax. Two surgeons, under the guidance of a nuclear medicine specialist, independently examined all PET images. The analysts were blinded to the pathology outcomes. Tumor SUVmax was calculated from the site of accumulation while referring to the results of enhanced CT and esophagogastroduodenoscopy to identify the lesion. The detailed methods are the same as those reported previously [15].

### 2.4 Surgical Procedure

The typical surgical approach for GC involved performing a gastrectomy along with D2 lymph node dissection. The surgical procedure for EGJC was proximal gastrectomy or total resection with lower or subtotal esophagectomy involving abdominal and mediastinal lymph node dissection. A Clavien‐Dindo classification was used to assess surgical complications (version 5.0).

### 2.5 Pathological Evaluation of Tumor Response

Two or more pathologists assessed the histological reaction of the tumor to NAC. An evaluation was conducted on the site where the tumor was found to be located during the pretreatment assessment. The grade of tumor response to NAC was classified on the basis of the number of viable cancer cells in the tumor based on the Japanese Classification of Gastric Cancer below: Grade 0, no effect (no evidence of effect); Grade 1a, very slight effect (Viable tumor cells occupy more than 2/3 of the tumorous area); Grade 1b, slight effect (Viable tumor cells remain in more than 1/3 but less than 2/3 of the tumorous area); Grade 2a, very moderate effect (viable tumor cells remain in less than 1/3 but more than 1/10 of the tumorous area); Grade 2b, moderate effect (viable tumor cells remain in less than 1/10 of the tumorous area); and Grade 3, complete response (CR, no viable tumor cells remain) [9]. Pathological CR was regarded as cases with ypT0 and ypN0.

### 2.6 Statistical Analysis

Results are expressed as medians and ranges and were analyzed for statistical significance using the Mann–Whitney *U* test. A *p*‐value of < 0.05 was defined as statistically significant. Overall survival (OS) and recurrence‐free survival (RFS) curves were constructed using the Kaplan–Meier method with the log‐rank test. To calculate the optimal cut‐off SUVmax for predicting histological tumor response, we performed receiver operating characteristic (ROC) curve analysis and measured the area under the curve (AUC). The optimal cutoff value was determined using the Youden index to maximize sensitivity and specificity. Statistical analyses were performed using the SPSS ver. 28 software (IBM Corp., Armonk, NY, USA).

## 3 Results

### 3.1 Patient Characteristics and Neoadjuvant Chemotherapy Findings

Patient clinical characteristics are shown in Table 1. Of the patients, 22 (73%) were male and had a median age of 72. Nineteen patients (63%) had tumors located primarily in the stomach, and over half (77%) were macroscopic type 1/2 tumors. The histology of the tumors was the predominantly differentiated type (66%). There were 28 cT3/4 cases (93%). None of the patients had any distant metastases.

**TABLE 1:** Patient characteristics.

Variable	*n* = 30	%
Age (years)	72 (34–84)	
Gender		
Male/female	22/8	73/27
Location		
Stomach/EGJ	19/11	63/37
Histologic type		
Pap/tub/muc/por/sig	1/19/0/10/0	3/63/0/34/0
Macroscopic type		
1/2/3/4	9/14/7/0	30/47/23/0
Clinical T status		
T2/T3/T4a/T4b	2/13/12/3	7/43/40/10
Clinical N status		
N0/N1/N2/N3	4/19/7/0	14/63/23/0
Clinical M status		
M0/M1	30/0	100/0

*Note:* Continuous data are presented as medians and ranges.

Abbreviations: EGJ, esophagogastric junction; muc, mucinous adenocarcinoma; pap, papillary adenocarcinoma; por, poorly adenocarcinoma; sig, signet ring cell carcinoma; tub, tubular adenocarcinoma.

The surgical findings and chemotherapy are summarized in Table 2. All patients received SOX therapy, with trastuzumab in two HER2‐positive patients. The standard number of three courses was performed in 90% of the patients. During chemotherapy, one patient developed a small nodule with suspected lung metastasis, and chemotherapy was continued for six courses. The nodule disappeared after chemotherapy, and the patient was considered amenable to radical resection. All patients underwent radical surgery after NAC. No chemotherapy or surgery‐related mortality was observed. All specimens taken from GC and EGJC radical resections were pathologically assessed for tumor reactivity. Three patients (10%) had pathological CR by NAC. Among the patients, 67% showed a histological response of Grade 1b or higher, while 43% showed a response of Grade 2 or higher.

**TABLE 2:** Treatment and pathological findings.

Variable	*n* = 30	%
NAC regimen		
S‐1 + Oxaliplatin	28	93
S‐1 + Oxaliplatin+Trastuzumab	2	7
Course		
2 cycles/3 cycles/6 cycles	2/27/1	7/90/3
Adverse events (CTCAE)		
All grade	15	50
Grade ≥ 3	3	10
Aspartate aminotransferase increased	1	3
Lung infection	1	3
Neutropenia	1	3
Surgical transition	30	100
Gastrectomy		
Distal/proximal/total	11/10/9	37/34/29
Esophagectomy		
Lower/subtotal/none	6/4/20	20/14/66
Number of resected lymph nodes	41 (8–90)	
Complications (CD Grade ≥ 2)		
Anastomotic leakage	3	10
Pneumonia	2	9
Bleeding	1	4
Intestinal obstruction	1	4
ypT status		
T0/T1/T2/T3/T4a/T4b	5/3/7/11/3/1	17/10/23/37/10/3
ypN status		
N0/N1/N2/N3	19/5/4/2	60/17/14/9
ypM status		
M0/M1	29/1	97/3
Histological tumor response (Grade)		
0/1a/1b/2a/2b/3	2/8/6/4/5/5	9/25/20/14/16/16
Pathological CR	3	10
Postoperative chemotherapy	13	43

*Note:* Continuous data are presented as medians and ranges.

Abbreviations: CD, Clavien‐Dindo Classification; CR, complete response; CTCAE, Common Terminology Criteria for Adverse Events; NAC, neoadjuvant chemotherapy.

### 3.2 Standardized Uptake Value in Positron Emission Tomography Before and After Neoadjuvant Chemotherapy

**FIGURE 1:**
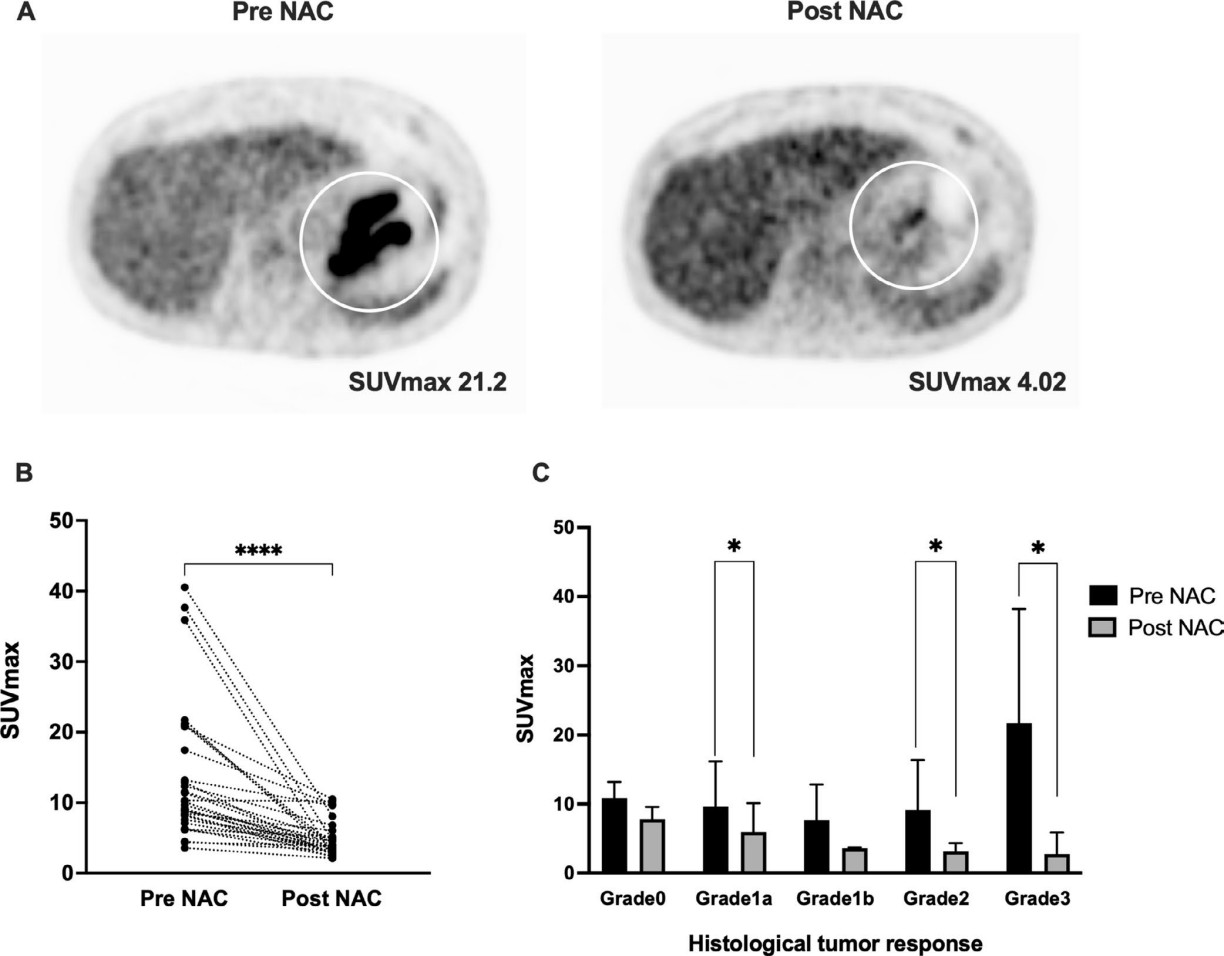
Comparison of SUVmax before and after NAC. (A) ^18^F‐fluoro‐2‐deoxyglucose positron emission tomography images before and after NAC in a 70‐year‐old man with gastric cancer (por, type2, cT3, N1, M0, SOX 3 cycle, and histological tumor response Grade 2b). (B) Comparison of changes in SUVmax values before and after NAC (*p <* 0.001). (C) Comparison of histological tumor response. There was a statistically significant difference between Grades 1a, 2, and 3 (*p* < 0.05). NAC, neoadjuvant chemotherapy; SOX, S‐1 + Oxaliplatin regimen.

The distribution of the SUVmax before and after NAC is shown in Figure 1A–C. The median SUVmax before NAC was 9.88 (3.59–40.53) and after NAC was 3.71 (2.11–10.52), which was significantly decreased (*p* < 0.0001). None of the patients showed an increase in SUVmax after NAC. The change in SUVmax according to histological response is shown in Figure 1C. The SUVmax decreased in all histological responses, with significant decreases in Grades 1a, 2, and 3 (*p =* 0.005, *p =* 0.025, and *p =* 0.023, respectively).

**FIGURE 2:**
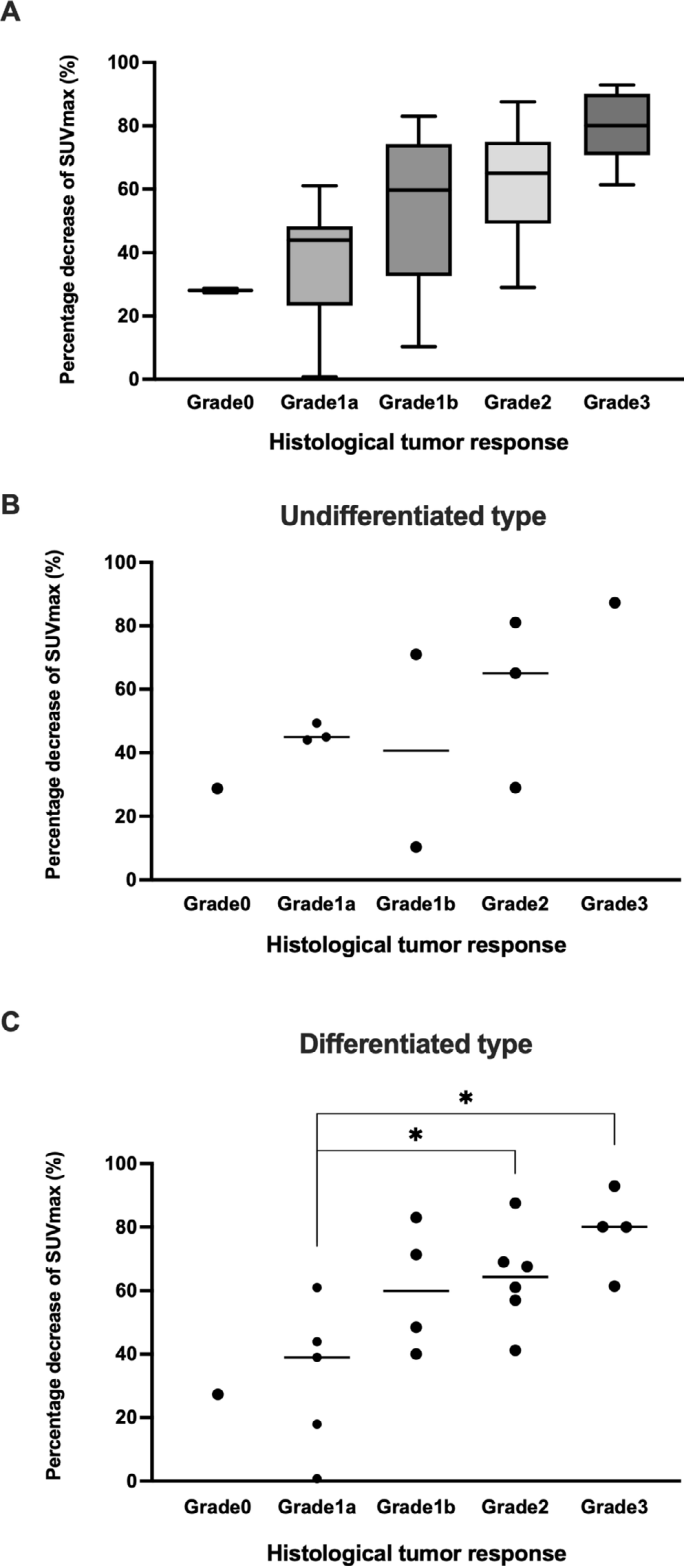
Correlation between the rate of decrease in SUVmax and histological tumor response. (A) Comparison across boards. (B) and (C) Comparison of the rate of decrease in SUVmax and histological tumor response according to histological type.

The correlation between the rate of decrease in SUVmax and histological response is indicated in Figure 2A. The median rates of decrease for Grade 0 and Grade 1a were relatively low at 28.1% (27.4–28.7) and 43.9% (0.78–61.0), respectively. In contrast, Grade 1b or higher exhibited progressively higher decrease rates: Grade 1b at 59.8% (10.3–83.0), Grade 2 at 65.1% (29–87.6), and Grade 3 at 80.1% (61.4–93.0). The rate of decrease in SUVmax by histological type showed a trend toward a stepwise increase with each histological response (Figure 2B,C). In the differentiated type, there were significant differences in the rate of decrease in SUVmax between Grade 1a and 2 (27.4 vs. 64.3, *p* = 0.03) and Grade 1a and 3 (27.4 vs. 80.1, *p* = 0.016). Among cases of undifferentiated type, there were two cases of Grade 1a or higher with a lower SUVmax reduction rate than Grade 0. The Grade 1b case was GC of the undifferentiated type without HER‐2 expression, and the Grade 2 case was EGJC of the undifferentiated type without HER‐2 expression (Figure 2B). Even in the differentiated type, two cases were found to have a lower SUVmax reduction rate than Grade 0. These two Grade 1a cases were GCs of the differentiated type without HER‐2 expression (Figure 2C). The two cases of HER2‐positive patients who received trastuzumab showed a reduction rate close to the median reduction rate for each grade (Case 1: papillary adenocarcinoma, SUVmax reduction rate 43.9%, histological response Grade 1a; Case 2: moderately differentiated adenocarcinoma, SUVmax reduction rate 69.0%, Grade 2a). There was no significant difference in the rate of reduction between GC and EGJC, but there was a trend toward a higher reduction rate in EGJC (GC: 49% [1–93] vs. EGJC: 65% [29–88]; *p* = 0.401).

### 3.3 Optimal Rate of Decrease in Standardized Uptake Value for Predicting Histological Response and Prognosis

**FIGURE 3:**
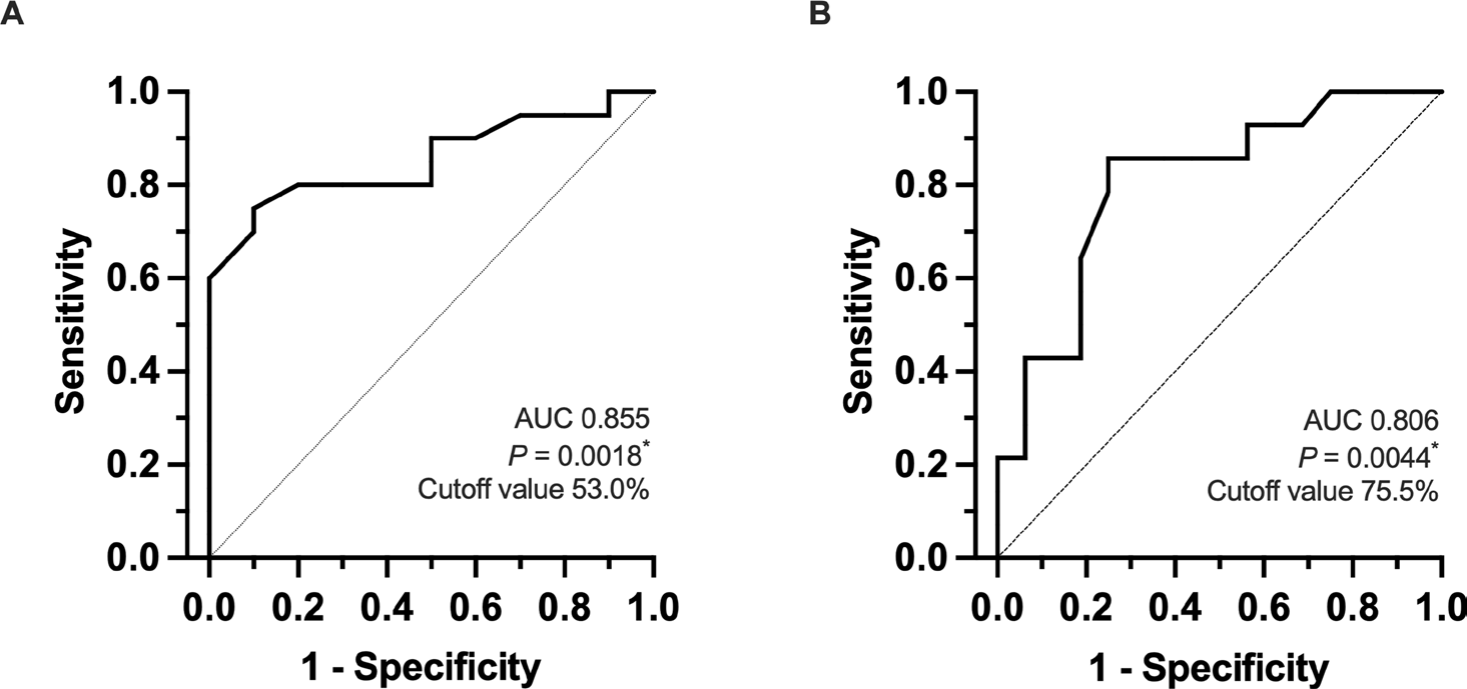
ROC analysis of the percentage decrease in SUVmax for predicting histological tumor response. The ROC curve demonstrates the optimal cut‐off value for each histological tumor response. (A) Grade 1b or higher, (B) Grade 2 or higher. ROC, receiver operating characteristics.

**FIGURE 4:**
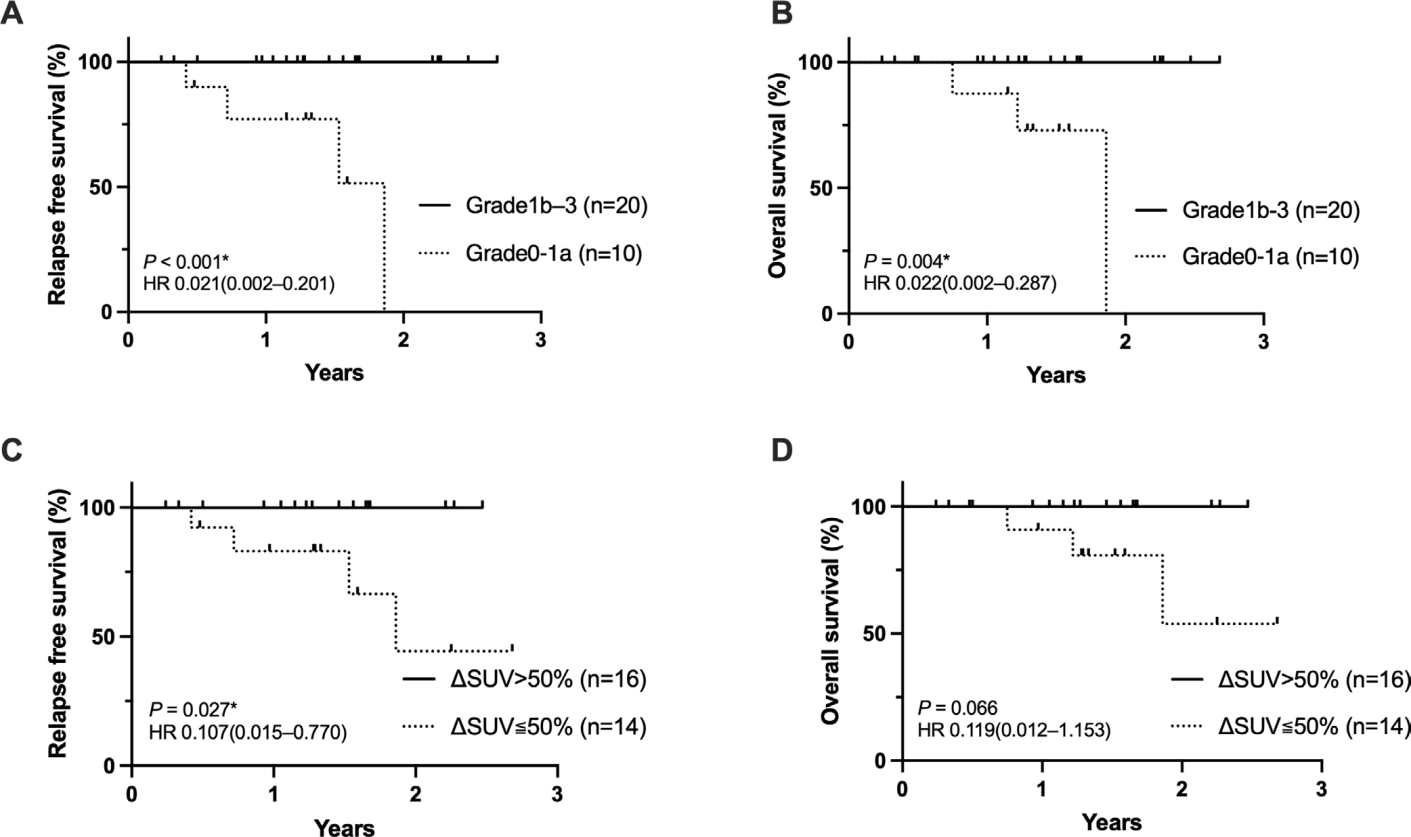
Relapse‐free and overall survival in patients with gastric and esophagogastric junction cancer by pathological tumor response (A and B) and percentage decrease in SUVmax (C and D). HR; hazard ratio.

ROC curve analysis was used to calculate the appropriate cut‐off value for the percentage decrease in SUVmax that predicts histological response. The optimal cut‐off value to predict Grade 1b or higher was 53.0% (*p* < 0.001, AUC 0.855; Figure 3A). A decrease of more than 3/4 in SUVmax before and after NAC was associated with histological efficacy determination of Grade 2 or higher (*p* = 0.004, AUC 0.806; Figure 3B). The group with Grade 1b or higher had a better prognosis for both RFS and OS (*p* < 0.001; HR, 0.021 [95% CI, 0.002–0.201] for RFS and *p* = 0.004; HR, 0.022 [95% CI, 0.002–0.287] for OS; Figure 4A,B). Decreases in SUVmax greater than 50% correlated with the histologic response of Grade 1b or higher and tended to have a favorable prognosis (*p* = 0.027; HR, 0.107 [95% CI, 0.015–0.770] for RFS and *p* = 0.066; HR, 0.119 [95% CI, 0.012–1.153] for OS; Figure 4C,D).

## 4 Discussion

In this study, the SUVmax of FDG‐PET in GC and EGJC showed a decreasing trend before and after NAC. FDG‐PET for GC and EGJC has not been reported to be useful in diagnosing tumor progression owing to low FDG accumulation, especially in the undifferentiated type [17, 18, 19, 20]. Our study indicated that FDG‐PET has the potential to be an indicator reflecting tumor viability changes due to chemotherapy at the primary site of GC and EGJC. The traditional method for evaluating the effectiveness of preoperative treatment is the Response Evaluation Criteria in Solid Tumors (RECIST) using the tumor diameter on CT scans [21]. The diameter of the primary tumors of GC and EGJC can be measured for large‐diameter lesions, such as advanced cancer. However, it is difficult to correctly assess treatment efficacy in some lesions that are difficult to measure, such as flat lesions with little elevation. In addition, the positive diagnostic rate of GC depth and lymph node metastasis by CT is low, and CT alone may not be sufficient to predict the efficacy of NAC [22]. In contrast, the SUVmax in FDG‐PET can measure tumor viability regardless of tumor diameter and localization. In evaluating the response to treatment with NAC for GC and EGJC, it may be useful to measure the change in SUVmax as well as RECIST for a more accurate assessment.

This study suggested that the percentage decrease in SUVmax before and after NAC for GC may correlate with histological tumor response and can serve as a prognosis predictor. Specifically, histological Grade 1b or higher represents a state where more than one‐third of proliferating cancer cells disappear. In our study, a reduction in SUVmax exceeding 50% is believed to reflect changes in the actual tumor environment [10]. Several previous reports on the rate of SUVmax reduction of NAC for GC and EGJC utilized “35%” as the criterion for identifying NAC response cases. These studies adopted this criterion to calculate the percentage decrease in SUVmax by performing a second FDG‐PET either after one course of NAC or on the 14th day following NAC initiation. Notably, this timing for calculating the percentage decrease differs from that adopted in our study, where calculations were performed at the first visit and before surgical resection (approximately 9 weeks) [23, 24]. Although these criteria have also been applied to examine the association of histopathological efficacy determination with the major response (equivalent to Grade 2b in this study), only a few reports depicted a significant correlation [24, 25]. Our study is the first to demonstrate a potential stair‐step correlation between the percentage of SUV reduction after completion of NAC and each histological response level. Notably, reductions of 75% or more predicted Grade 2 or higher. If the SUVmax decreases by less than 50% after preoperative chemotherapy, changing the chemotherapy regimen without proceeding to surgery may be an option, as the effect is judged to be weak. Conversely, if a reduction rate of 75% or higher is achieved, surgery may potentially be avoided in elderly patients. Several reports, including our previous report on NAC in GC and EGJC, have shown an association between histological tumor response and prognosis, with Grade 1b or higher showing a particularly favorable prognosis [26, 27]. The present cut‐off value for the percentage of SUVmax reduction could be used for NAC of GC and EGJC to predict not only detailed preoperative treatment efficacy but also prognosis.

Perioperative chemotherapy with FLOT is the standard therapy for GC and EGJC in Western countries. However, in Asia, where the incidence is high, clinical trials of perioperative chemotherapy such as SOX and DOS using S‐1, an oral anticancer drug, are widely conducted. Although some studies have demonstrated the usefulness of PET in predicting the efficacy of perioperative FLOT therapy, there has not yet been a comprehensive study of SOX therapy, representing a novel aspect of our study [28]. Based on the present results, measuring SUVmax on PET for GC and EGJC in various chemotherapy regimens is a promising predictor of treatment response.

The present study has several limitations. It was a nonrandomized retrospective examination conducted at a single institution with a small patient cohort. The patients in this study included those with GC and EGJC, which exhibit different biological behaviors. The cut‐off value was determined using ROC curve analysis; however, cross‐validation was not performed. Multivariate analysis such as Cox regression was not conducted due to the small number of samples and events. Some cases had a follow‐up period of less than 1 year. Additionally, this study did not evaluate tumor response using CT imaging. These data are preliminary, and future multicenter studies with larger and more diverse populations are required to address the potential impact of demographic factors. We consider the preliminary findings of this study to serve as a stepping stone for future research on the effectiveness of PET.

In conclusion, FDG‐PET may be a promising predictor of treatment response, as it appears to correlate with tumor viability before and after NAC for GC and EGJC. An optimal value for the rate of decrease in SUVmax could allow for a more detailed prediction of histological tumor response and prognosis.

## 5 Author Contributions

All authors had full access to the data in the study and take responsibility for the integrity of the data and the accuracy of the data analysis. Conceptualization, N.U. and S.K.; Methodology, N.U. and S.K.; Investigation, N.U., R.S., Y.K., and T.I.; Formal Analysis, H.H., H.G., and T.M.; Resources, N.U., H.H., and K.Y.; Data Curation, N.U. and T.I.; Writing – Original Draft, N.U. and S.K.; Visualization, N.U.; Supervision, Y.K.

## Conflicts of Interest

The authors declare no conflicts of interest.

## Data Availability

Research data are not shared.
